# First Detection of a Novel Posavirus 2 Strain Identified from Pigs in China

**DOI:** 10.3390/pathogens13121036

**Published:** 2024-11-24

**Authors:** Li Chen, Haohao Lu, Xue Gao, Han Zhou, Jinghao Wang, Zhidong Zhang, Bin Chen, Chun Li, Luqi Liang, Long Zhou, Yi Zhang

**Affiliations:** 1 College of Animal and Veterinary Sciences, Southwest Minzu University, Chengdu 610041, China; chenli@stu.swun.edu.cn (L.C.); luhaohao@stu.swun.edu.cn (H.L.); gaoxue@stu.swun.edu.cn (X.G.); zhouhan@stu.swun.edu.cn (H.Z.); wangjnghao@stu.swun.edu.cn (J.W.); zhangzhidong@swun.edu.cn (Z.Z.); 2 Key Laboratory of Veterinary Medicine in Universities of Sichuan Province, Southwest Minzu University, Chengdu 610041, China; 3 Sichuan Provincial Center for Animal Disease Control and Prevention, Wuhou District, Chengdu 610041, China; 18244245137@163.com (B.C.); lich03@163.com (C.L.); liangluqi@126.com (L.L.)

**Keywords:** Posavirus 2, China, complete genome, phylogenetic analysis

## Abstract

Porcine stool-associated RNA virus (Posavirus) is an unclassified virus with sequence similarity to viruses in the order of Picornaviridae. In China, lineage 1 Posavirus (Posavirus 1) has been circulating in the field since its initial emergence in 2014 however no other lineages have been reported. To investigate the genetic diversity of Posavirus in China, 1200 diarrheic samples were collected from pigs in China. Following high-throughput and Sanger sequencing, one complete genome sequence of a Posavirus (designated SC01) strain was obtained. The genome of SC01 strain was 10217 nucleotides in length and encoded a polyprotein of 3346 amino acids. Genome comparative analysis revealed that SC01 shared 85.6% nucleotide similarity to Posavirus 2 strains, but only 35.2–58.0% sequence identity with Posavirus 1 and 3–12 strains. Phylogenetic analysis showed that the SC01 was classified in Posavirus 2 and clustered into a separate branch with the American Posavirus 2 isolates, indicating that the SC01 is a Posavirus 2 strain. Notably, a novel 1-amino acid deletion was observed in polyprotein at amino acid position 147. This is the first report of the presence of Posavirus 2 in China, and the genomic data of SC01 provides insights into the genetic diversity and evolution of Posavirus in the region.

## 1. Introduction

Porcine viral diarrhea disease is the most common and costly problem in pigs that substantially increases the mortality and morbidity of these animals, contributing to enormous economic losses in the swine industry [1]. Currently, several viral pathogens can cause serious intestinal disease in swine, such as the well-known porcine epidemic diarrhea virus (PEDV), porcine transmissible gastroenteritis virus (TGEV), porcine delta coronavirus (PDCoV), and porcine rotavirus (PoRV) [2]. Recent studies have suggested that many enteroviruses, which belong to *Astroviridae*, *Caliciviridae*, *Coronaviridae*, and *Picornaviridae*, are potential pathogens associated with diarrhea in pigs [3]. 

Porcine stool-associated RNA virus (Posavirus) is a single stranded positive-sense RNA virus, and it is classified as the novel member of the order *Picornavirales*. Posavirus was first identified in the feces of piglets with diarrhea in the USA in 2011, and it was subsequently detected in the feces of healthy piglets and adult pigs [4,5,6]. To date, Posavirus was confirmed in South Africa, Japan, United States, South Korea, Belgium, Germany, and China, suggesting that the virus is widely spreading around the world [7,8,9,10]. The following twelve distinct lineages of Posavirus have been identified worldwide: Posavirus 1–12 [9,11]. However, Posaviruses 1 and 3 were detected the most [9]. Posavirus 1 was reported in the seven countries mentioned above, and Posavirus 3 was reported in both the United States and South Korea [4,6,11]. At present, Posavirus 2 has only been reported in the United States [4]. In China, Posavirus 1 was first identified in the feces of diarrheal and healthy piglets by viral metagenomics in 2014 [8]; however, since its initial emergence in 2014, no other lineages have been reported in China.

The viral metagenomic technique is an effective strategy for investigating unknown etiologies and identifying uncultured viruses. In recent years, this technique has been widely used in the analysis of porcine enteroviruses, and some new viruses associated with diarrhea have been identified in domestic pigs [7,8,12,13]. This study aims to detect and characterize Posavirus using a viral metagenomics approach to discover potential new lineages in Chinese swine populations. Additionally, we analyzed the obtained gene sequences to determine their phylogenetic relationships with known lineages. Through this study, we expect to deepen our understanding of the epidemiology of Posavirus in China.

## 2. Materials and Methods

### 2.1. Sample Collection and Treatment

To investigate the genetic diversity and variation in Posavirus in China, 1200 fresh rectal fecal samples of diarrheic piglets (1–3 months of age) were collected from 63 farms in the area of Sichuan Province, China. The samples were pretreated before RNA extraction. In addition, fecal samples were vortexed and centrifuged. For each piglet fecal sample, 100 μL of supernatant was collected and mixed into a pool (*n* = 1200/pool). The pooled samples were then passed through 0.22 μm filters (Millipore, Burlington, MA, USA), and an Ultra 50K ultrafiltration tube (Millipore, USA) was used to concentrate the resulting filtrates. The 2 mL filtrates were incubated with a cocktail of nucleases (DNase and RNase enzymes) for 2 h at 37 °C. Total viral RNA was extracted, and reverse transcription was performed using random hexamers and the Superscript III RT reverse transcriptase kit (Invitrogen, Carlsbad, CA, USA). 

### 2.2. Library Construction and High-Throughput Sequencing

To construct the cDNA library, the obtained cDNAs from clinical samples were ultrasonicated into small fragments (about 350~500 bp). One paired-end library was constructed by DNA fragments end repair and adaptor ligation. Then, the prepared library was sequenced using Illumina’s NovaSeq 6000 platform (Illumina, Shanghai, China), and the sequencing depth in the study was 10 G. The Illumina-generated raw sequences were filtered using Trimmatic software (v0.36) to trim adaptor-related reads, low-quality reads, reads with a high proportion of N-bases (>10%), and short-length reads (<75 bp), resulting in high-quality clean data. The cDNA library construction and high-throughput Illunima Novaseq sequencing of the above samples were performed by TP-Bio Co., Ltd. (Shanghai, China). The Illumina-generated reads were de novo assembled using Megahit software (v1.0) (HKU-BGI, Hong Kong, China). The assembled viral contigs were aligned with sequences using BLASTn and BLASTx in the NCBI database. The taxonomies of the viral sequences with >90% overlap identity were selected and used for grouping and analyses. The viral abundances were calculated by SOAP aligner software (v2.0). To confirm the genomic sequences of Posavirus, 5 pairs of PCR primers were designed based on the sequences obtained from high-throughput sequencing (Supplementary Table S1). The cDNA was amplified using high-fidelity polymerase Platinum SuperFi II DNA (Invitrogen, USA). The amplified PCR products were then purified and cloned into the pMD19-T vector (TaKaRa, Dalian, China). Three positive clones from each fragment were subjected to the commercial service (Sangon, Shanghai, China) using Sanger sequencing. 

### 2.3. Sequence Comparison and Phylogenetic Analysis

Sequence assembly and multiple sequence similarity analyses were performed using the SeqMan and MegAlign program of DNASTAR 7.0 software (DNASTAR Inc., Madison, WI, USA), respectively. Multiple sequence alignment and phylogenetic analysis were performed with the MEGA X software (v10) to build a maximum-likelihood phylogenetic tree with the General Time Reversible model (1000 bootstrap support). To analyze the genomic characterization of the novel SC01 strain with other Posavirus lineage 1–12 strains, a total of 45 complete or near complete genomes (>7900 nt) of Posavirus strains were downloaded from the database of the National Center for Biotechnology Information (NCBI). Recombination events were assessed using Recombination Detection Program 4.0 (RDP 4.0, version 4.96).

## 3. Results

Metagenomics analysis identified eleven distinct viruses belonging to five taxonomic families in the pooled library, with *Picornaviridae* being the main member, accounting for 45.57% (Supplementary Figure S1). The largest contig of *Picornaviridae* was 10217 bp and showed the highest nucleotide identity with the American Posavirus 2 strain. The complete genome of the Posavirus strain was confirmed by RT-PCR and the virus was designated as SC01. The sequences were submitted to GenBank under the accession number PP795245. The length of the SC01 strain’s complete genome was 10,217 nucleotides. Similarity analyses showed that the genome of SC01 shared the highest nt identity (85.9%) and polyprotein aa identity (96.2%) with two American Posavirus 2 strains. However, the new partial genome of SC01 showed lower identity, in terms of nt (35.2–58.0%) and aa (13.3–68.9%), compared to reference genomes of Posavirus 1 and 3–12, respectively (Table 1).

**Figure 1:**
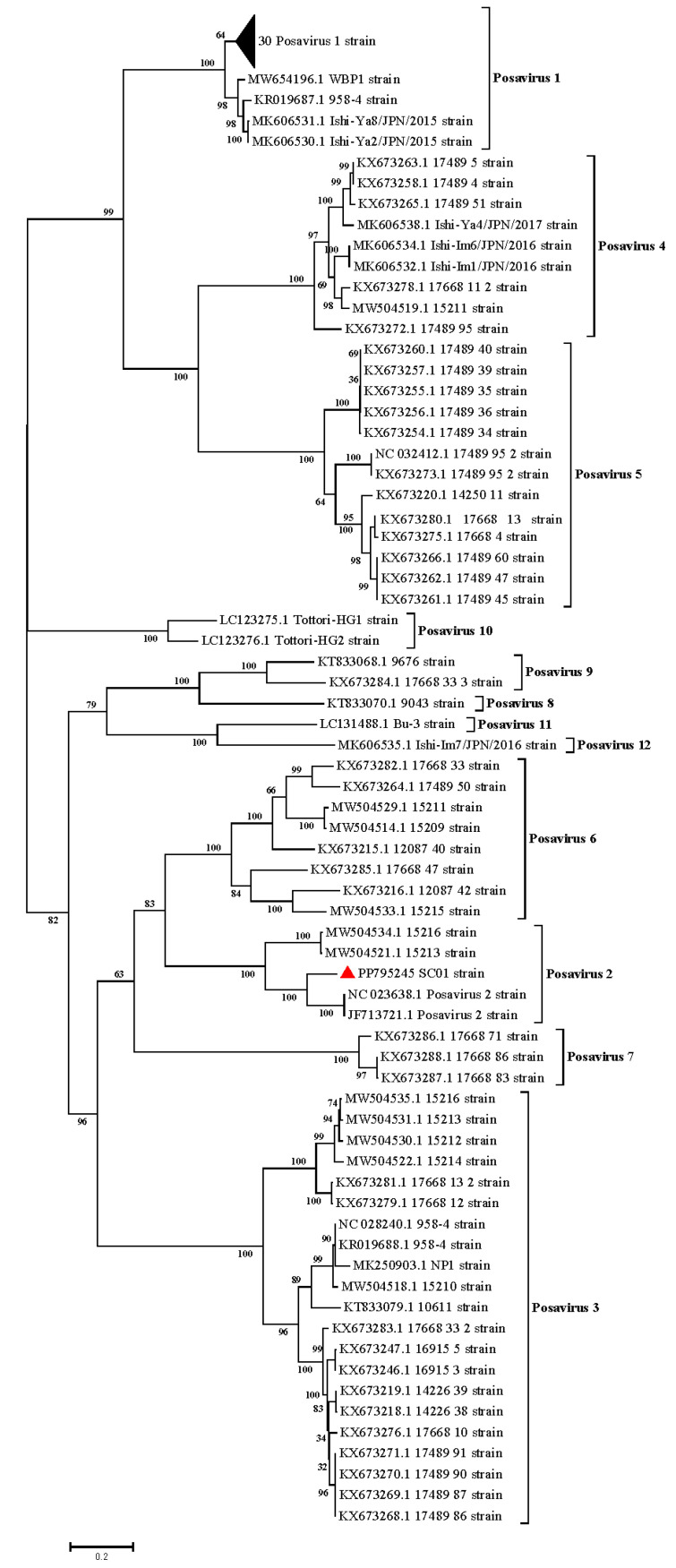
Phylogenetic analysis of Posavirus lineage 1-12 based on the RNA-dependent RNA polymerase (RdRp) region. ▲ represents the Posavirus 2 sequence of SC01. The numbers along branches are bootstrap values. The scale bar indicates the number of nucleotide substitutions per site.

Posavirus lineages were determined based on complete genome phylogenetic analysis. All of the 100 reference Posavirus strains can be divided into the following twelve lineages: Posavirus 1-12. SC01 strain genomes formed a relatively distinct branch within the Posavirus 2 lineage in the phylogenetic tree (Figure 1). These results indicated that the SC01 strain belongs to the Posavirus 2 strain and has unique evolutionary characteristics.

**Figure 2:**
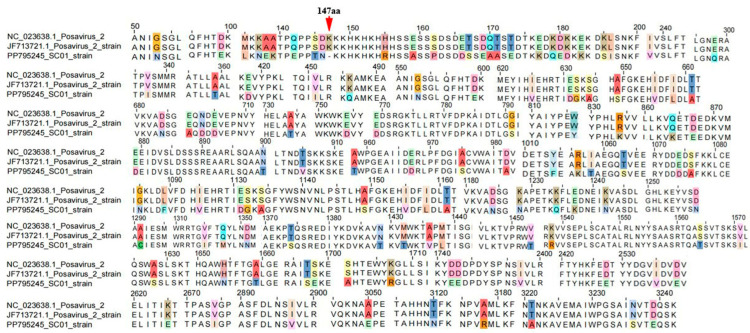
Location of the mutation site of the Posavirus 2 SC01 strain. The mutations sites are indicated with colored boxes. One amino acid deletion at position 147 of the polyprotein was marked with a red arrow. Single-letter abbreviations represent the amino acid residues.

At the aa level, the reference genomes of Posavirus 2 strains (NC_023638.1 and JF713721.1) encoded a total of 3347 aa, and the SC01 strain encoded 3346 aa. Based on the reference genomes, the aa similarity of the SC01 isolate to the Posavirus 2 strains was 95.7%. All of the Posavirus 2 strains encoded a polyprotein. The SC01 strain had a single aa deletion (position 147aa) and 142aa mutations (Figure 2), with a mutation rate of 4.27%.

**Figure 3:**
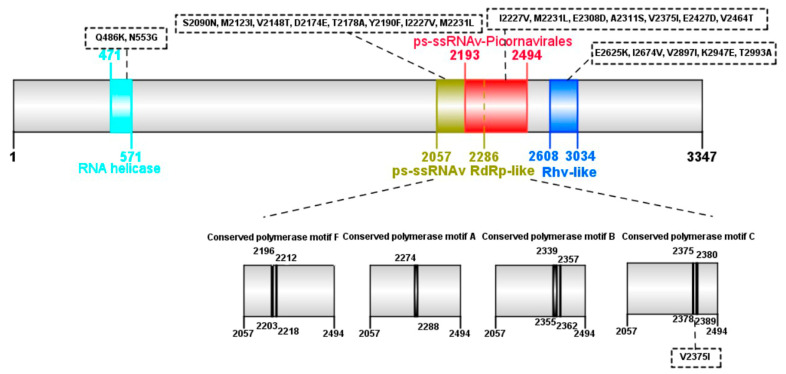
Distribution of the Posavirus 2 SC01 strain mutations in the replicase polyprotein domains (an annotation of the Posavirus 2 replicase polyprotein domains structural from NCBI). The positions of the mutation sites are indicated by dashed boxes. The RNA helicase is indicated by the cyan region (position 147–157), the ps-ssRNAv RdRp-like domain is indicated by the green region (2057–2286), the ps-ssRNAv-Picornavirales domain is indicated by the red region (2193–2494), the Rhv-like domain is indicated by the blue region (2608–3034), and the conserved polymerase motif domain is indicated by the gray region (2057–2494).

The present study explored the mutation sites located in important protein structural domains. Figure 3 depicts a protein structure map based on the Posavirus 2 reference protein annotations in the NCBI database (NCBI Reference Sequence: YP_009010972.1), and the positions of the mutation sites are indicated by dashed boxes. A total of 20 aa mutations were identified in the protein structural domains, with two aa mutations in the RNA helicase, eight aa mutations in the ps-ssRNAv RdRp-like domain, seven aa mutations in the ps-ssRNAv-Picornavirales domain, five aa mutations in the Rhv-like domain, and one aa mutation in the conserved polymerase motif C domain.

## 4. Discussion

The presence of Posavirus in pig populations was reported in several countries, suggesting that the virus is widely distributed [4,5,9,10,11]. In the relevant literature, the presence of Posavirus was detected by using genotype-specific RT-PCR methods [4,11], and different countries were found to have different positive rates. For example, Posavirus 1 was detected in 3.4% (5/149) of the swine fecal samples collected in China [11]; whereas in the samples collected in the United States, the rate was 47.2% (17/36) [4]. To the best of our knowledge, this is the first time that the genome of Posavirus 2 has been obtained in China, providing important data to support the development of detection methods. 

In the Posavirus lineages 1–12, the complete genomes ranged in length from 8887 to 10,220 nt and the genomes encoded polyproteins, ranging from 2942 to 3347 amino acids. A complete genome phylogenetic tree based on Posavirus 1, 2, and 3 showed that the SC01 strain genome formed a relatively distinct branch in the Posavirus 2 in the phylogenetic tree. These results suggest that the SC01 strain belongs to the Posavirus 2 strain with unique evolutionary features. Further sequence analysis revealed that the SC01 strain had a single aa deletion (position 147aa) and 142aa mutations. However, at 143 amino acid variants, we found a total of 20 aa mutation sites located within structurally important domains of the protein, including the following: RNA helicase, ps-ssRNAv RdRp-like domain, ps-ssRNAv-Picornavirales domain, Rhv-like domain, and Conserved polymerase motif C domain. Conserved features shared by all Picornavirales include the helicase, chymotrypsin-like protease, and RNA-dependent RNA polymerase (RdRp) structural domains, which are important for viral replication [14]. Currently, as Posavirus cannot be isolated in vitro, its pathogenicity, replication, and pathogenesis remain unknown. Thus, more efforts have been concentrated on viral detection, genetic characterization, and phylogenetic analyses. Since Posavirus was firstly discovered in 2010 [4], many posaviruses and posa-like viruses have been detected in humans [15] and vertebrates such as fish [16], rats [17], bats [17], giant pandas [18], and gorillas [19], implying a broad species host tropism with a high risk of interspecies transmission. Therefore, the viruses deserve continuous monitoring, and the biological significance of these variants warrants further investigation.

In summary, a novel Posavirus 2 strain, SC01, with unique genetic variation within its genome was identified in China for the first time. Phylogenetic analysis showed that the SC01 isolate was clustered into Posavirus 2 with a separate branch, indicating a unique genetic evolutionary trend. Additionally, 1 novel aa deletion and 142 mutations occurred at the aa level. The genomic data of SC01 enriches the epidemiological and genetic evolutionary information of Posavirus 2 in China.

## Figures and Tables

**Table 1:** Nucleotide and amino acid homology between SC01 strain and 45 reference strains.

Lineages	Strains	Country/Year	Accession No.	nt Identity (%)	Polyprotein aa Identity (%)
Posavirus 1	Belgium/01/2019	Belgium/2019	MT642667.1	36.0	24.3
	BSF3	South Africa/2021	OM104037.1	36.4	24.3
	BSF2	South Africa/2021	OM105049.1	36.6	68.6
	Ishi-1	Japan/2015	LC123281.1	36.1	68.0
	Iba	Japan/2015	LC123280.1	36.4	68.2
	Iba 26-487	Japan/2015	LC123279.1	36.4	68.4
	9754	USA/2015	KT833064.1	36.4	24.1
	HBTS-11	China/2015	KU981058.1	36.2	68.1
	SDQD-25	China/2014	KT724282.1	36.5	68.2
	WBP1	China/2015	MW654196.1	37.1	68.9
	SS 2017	South Korea/2017	MN090139.1	36.2	68.4
	NP1x 2018	South Korea/2018	MN090138.1	36.3	68.0
	DTx 2019	South Korea/2019	MN090137.1	36.1	68.4
	Ishi-Im8/JPN/2016	Japan/2016	MK606536.1	36	68.4
	Ishi-Im2/JPN/2016	Japan/2016	MK606533.1	36	68.4
	Ishi-Ya8/JPN/2015	Japan/2015	MK606531.1	36.8	68.6
	Ishi-Ya2/JPN/2015	Japan/2015	MK606530.1	35.2	68.7
	Ishi-Im3/JPN/2015	Japan/2015	MK606529.1	36	68.4
	Ishi-Im1/JPN/2015	Japan/2015	MK606528.1	36.1	68.4
	958-4	USA/2015	KR019687.1	36	68.5
	PsaV-GER-L01017-K01-15-07-2015	Germany/2017	LT898419.1	35.8	68.6
	Belgium/02/2019	Belgium/2019	MT642668.1	36.0	24.3
	Posavirus 1	USA/2010	NC_023637.1	36.1	67.8
	Posavirus 1	USA/2010	JF713720.1	36.1	67.8
Posavirus 2	Posavirus 2	USA/2010	JF713721.1	**85.9** ^1^	**96.2**
	Posavirus 2	USA/2010	NC_023638.1	**85.9**	**96.2**
Posavirus 3	10611	USA/2015	KT833079.1	35.6	13.3
	958-4	USA/2015	KR019688.1	43.5	67.1
	NP1	South Korea/2018	MK250903.1	43.2	67.3
	958-4	USA/2015	NC_028240.1	43.5	67.1
Posavirus 4	Ishi-Ya4	Japan/2017	MK606538.1	39.9	27.1
	17668-11	Viet Nam/2013	KX673278.1	40.3	27.2
Posavirus 5	17489-40	Viet Nam/2013	KX673260.1	38.6	25.5
	14250-11	Viet Nam/2012	KX673220.1	38.6	25.3
Posavirus 6	12087-40	Viet Nam/2012	KX673215.1	58.0	32.2
	15215	USA/2018	MW504533.1	57.2	31.1
Posavirus 7	17668-86	Viet Nam/2013	KX673288.1	49.1	25.3
	17668-71	Viet Nam/2013	KX673286.1	49.0	25.3
Posavirus 8	9043	USA/2015	KT833070.1	45.4	28.2
Posavirus 9	9676	USA/2015	KT833068.1	44.4	24.3
	17608-33-3	Viet Nam/2013	KX673284.1	45.3	26.0
Posavirus 10	Tottori-HG1	Japan/2015	LC123275.1	41.6	28.9
	Tottori-HG2	Japan/2015	LC123276.1	42.2	27.6
Posavirus 11	Bu-3	Japan/2016	LC131488.1	45.3	24.6
Posavirus 12	Ishi-Im7	Japan/2016	MK606535.1	42.2	24.3

^1^ The highest nucleotide identities of different regions are indicated in bold typeface.

## Data Availability

The genome sequences of SC01 have been deposited in the GenBank database (23-Sept-2024) under the accession number of PP795245.

## Supplementary Materials

The following supporting information can be downloaded at: https://www.mdpi.com/article/10.3390/pathogens13121036/s1, Table S1: Specific primers used to amplify the genome of Posavirus 2 strain SC01. Figure S1: Family (a) and species (b) classification and percentage of virus sequences detected from diarrheic fecal samples from pigs.
