# Supplementary material for: First Detection of a Novel Posavirus 2 Strain Identified from Pigs in China
Source: Pathogens. 2024 Nov 24;13(12):1036. doi: 10.3390/pathogens13121036 (PMC11728614; doi:10.3390/pathogens13121036)
Supplement: Supplementary file 1 [file pathogens-13-01036-s001.zip › Supplementary_Material 1.pdf]

Supplementary Material

1 Supplementary Figures and Tables

1.1 Supplementary Tables

Table S1. Specific primers used to amplify the genome of Posavirus 2 strain SC01.

Virus	Primer name [a]	Primers sequence (5′-3′)	Primer position	Length (bp)
1	SC01-1F	TCCCGTCAGGGTAGATTTA	1-19	2292
	SC01-1R	TCTATGTCTTCCCGCTTG	2270-2292	
2	SC01-2F	CTAGCACCAAGCGGGAAGA	2268-2287	2081
	SC01-2R	CCCATCGGCACTAGACCA	4331-4348	
3	SC01-3F	TCCGAGGATAAGGAGAAG	4286-4303	2093
	SC01-3R	AGACCTGGATTACTGTTGA	6360-6378	
4	SC01-4F	GCGTGTTCACATCCCTTCA	6214-6232	2317
	SC01-4R	CAACGAATCCGCCAAGTC	8513-8530	
5	SC01-5F	ATGATAACACGCTTTACACCT	8202-8222	1984
	SC01-5R	ACATTAGGTCCGGATAATGCT	10165-10185	

[a] F and R represent forward and reverse primers, respectively.

Figure S1:

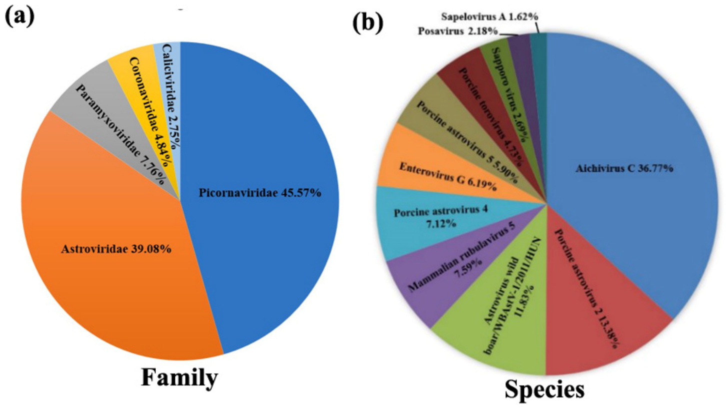

Figure S1: Family (a) and species (b) classification and percentage of virus sequences detected from diarrhoeic fecal samples from pigs.

Using high-throughput sequencing and BLASTx software, members of five virus families were identified, which, in order of sequence read abundance, were as follows: *Parvoviridae* (45.57% of all reads), *Astroviridae* (39.08%), *Paramyxoviridae* (7.76%), *Coronaviridae* (4.84%), *Caliciviridae* (2.75%). Eleven distinct viruses were identified. In order of sequence read abundance, the viruses were as follows: Aichivirus C (36.77% of all reads), Porcine astrovirus (13.38%), Astrovirus wild (11.83%), Mammalian rubulavirus 5 (7.59%), porcine astrovirus 4 (7.12%), Enterovirus G (6.19%), porcine astrovirus 5 (5.90%), porcine torovirus (4.73%), Sapporo virus (2.69%), Posavirus (2.18%), and Sapelovirus A (1.62%).
