# Supplementary material for: First Detection of a Novel Posavirus 2 Strain Identified from Pigs in China
Source: Pathogens. 2024 Nov 24;13(12):1036. doi: 10.3390/pathogens13121036 (PMC11728614; doi:10.3390/pathogens13121036)
Supplement: Supplementary file 1 [file pathogens-13-01036-s001.zip › Supplementary_Material 2.pdf]

Supplementary Material

1 Complete genome sequences of Posavirus SC01

TCCCGTCAGGGTAGATTTACGCTTCTTGCCGACCTTCGACGACTCGTTCCGACCACCGGA
AGACGTTGGTTATAACGCCTTGGTAGGGGAAGTCCTGTATCGCAAGTTACGTTTGCCTAG
GTATATGCATCCCACTACGTGGACAACACAGTTTATAGCATGCATAGTTAATTGGTTGAA
CCCCCTGTACTATTTAGTACTCCTCCAGCAAACTATAGAGTATGGATCTTATGTGGAAGT
TGATGACACCATTGAGGACCGACATAATGATTATTGGCCTCGTTTAGCAGATGGGTTTAT
TCACCACAAGCATGTGACCTTGTCACCCCGAGAGTATGAGCATTTTATTGATCGAATGAC
TGAATGTCAAGCTCAGAATACACAAGCTGCTGGATGGATTTCGTACTTGTTTACTAGTCT
GACGGACTATATAATTAACGAAACCGAATTTGGCGACAATGATCTCACTTATGAAGACA
TTGTATATGAAATGGAATTTGAGAGCCAGGGCGTTGGTTCATCCAAATTAAAGAATGAG
AAGACTCCTGAGCCACCCACTAATAAGAAGCACAAACATAAACATCGACATTCCAGCGC
GTCGTCCCCGGACAGTGATGACTCCTCTGAAGCCGCTTCTGAGGATACAAAGAAGGACC
AAGAGGACAAGAAGAAAGAGAAAGGCTTCTTTAAACGTGCATTTGATAGTATATCTAAT
AAGTTCTCTAGCATGATTGATACCGTCACAGATAAAACAAAGGAAGCAGCTTCTGACAT
CGCCCAAAAATCTATCATGAAAACTATACGTAGTATAGCTTCGAGCATATGGAGCAGTG
TCGTTTCACTATTTACAGGAGTGATGCAGTACCTCCGCGCACATGGAGTCCGCATGTTTA
TTGCATTACTTGCACTCAGTCTCATTTATGGCCTTGCCTATATTATAGTCGGACGCAACG
CCTTAGACAATACGTTCTGTCGTGCCCTTTTTGCTCCACTTAACAAGCTCCTAGGCAACC
AGCGTGCCTTTTCTGAAGCCATTGAGGCCCAAGGTATGGAACCCGTCCCTGCTCTCCTAG
CCACCATTGCTGCTCCTATAGCTGTATCTAGCTCAACTACCTACCACTCCACCCCTATCTC
AATGATGCGGGATTATTGCACTGCTATTTCTGCCATCTCTTCTGGCTCTGCTACGCTATTG
ACCGCCTTAAAAGTCCTGCCAGCTGGTTTAGCCTCTTTCTTCGAAAAGTGGTTCGATCCA
GAGCACAAGATAACTAAAGGAGAGACTGATGCTCTTGAATACGCTATACAGCAACTCTA
TAGTGTCATTAATTCTCCTGCTATGCTAGCCTCCCCGGGATTTGCAAAGTATATCAAAGA
TGTATACCCCAAGTTAGACGCGATGGTTAAGTATTCAACCCTTAGCGCGCAAGCTCTTAT
GCAATGGCGAGAGGTAGCTTCCATCTATACACAGATTATCCTTAGGGACACTGCAGGAA
CCTTCAGGTTTGAGCCTTACTACGTCCATGTGGCTGCTTCGCCTGGTGCCGGTAAGTCGC
TTTTCACACCACATATCCTCAAGCAAGCAATGAAAGAAGCCACAGGAAAGGACCCATCC
TTTAAAGCCCTAGGATTAGATCTTCTTGGCTACGCTGGTGAGGATATCATAATGATTGAT
GAGTTTGCTACCACTACAGTGTCTGCCAAAGACATGGCCAATTTATTGACTATCGTTTCA
TGTCTACCCAAGAACGTATCCAATAACCCATCACTGGCAAACATCAATTCAGGGCTGAA
AGGTATAAGCGTCATTCCCACGATTGTCTGCACCTCTGGTAACTTTGTGAACGTATCAAT
GCCTACTGATGAGATAGACAGGGCCTGGAAGAGAAGAATGAATTGTCAGATCCAGTTCC
ATACGGAGAAAGCCATTAATCTAACTGGCTGTGAGACCATAGAAGATATACAGAACGCT
TTTGGTGAGCAGGTCGAAATTGAGGTCTGGTTCCAACGAGGGTCCGTTACACAACGCCC
TGAGACTGTCACTGACTTGGTTACCTTACTGGCTGCTGATCTGCATAACAAAATGGAACG
TCATAAAGTTATGAAGAAAACATTTGGCTATGATGATGTTGAAGATGTTGCCCGTGACA
CTTTGAATAGGCTGACCAGTACTCTAGATAGTACAATCAAGACCCTTGATGCTGCCATCC
AACGACTTCCTGTTGCCCAGGATGATGACGTCGAACCTAATGTTTATGTTCCAGATAGAA
CTGACACCAAGCGGGAAGACATAGAAGACTTCCTGGTAGAACATGAGCTAGCAACTTAT
GCCGGCAACTTTGATATTGGCTTCTACCGCGTTCGTACAAACTGGAAATGGAAGGATGT
CTACGATTCTGGTACCGACCCAAATACGATTATTGATTTTGATGATTCACGTGGCAAGAC
CTTGCTTCGAACCGTCTTTGACCCGAAAGCCATTGACACCTTGGGTAGCATTTTAGACTC
TGACCCCGACGCCTATAAGTGGTCCTTCCTTATTAAGTACGTCTATGCCATCTACCCTGA

ATATGCACGAAAGAAGGGCTATACTCGTAAGCCGCCTATTTTCTATCCTCATCTTAAGGT
TGTTGCAGGTTATTGCTTCCCATGCCTCGAGGATGCGGCCAAATGTTCTTCCGACCCCGC
TAAAGCCTCTGCTTTGCTCAAAGTCAAGGAGACCGAGGAAGACAAGGTAATGCCAATTC
TTTACGAGCCTGATGAGATTGATGTATCACTAGATAGTTCTTCACGTGAAGCTGCACGAT
TGTCGCAAGCTGCTACGCTCACCAATGATGTATCTAAGAAAAGCAAAGAAGAACAGGA
GGAAGATTTAGATGCCATTAATAAGGCCATTCATGATCGATGGGTTGAGGAAGGCGCGC
TTGGATCCGCCCCTCCTAAGTTTAAGTCTATGTCTAAGTATTATGATTCCCTTCTGGTCAA
GCCTGTTAAAACTTGGCCTGGTGAGGCTATTATCGATGATCGCCTCCCTTTTGATGGTAT
CTCGTGTGTCTGGGCAATCACTGCAGTTAATCCCGACAGGGTCGTGGGTAGTGGTGTTGC
AACTTACAAATATCCCTATGCAGGTGTTAGGCTTTACTTCATAGGTGATGAGACTTCTTT
TGAAGCGAACCGCTGCTTCGATTCCGCTATGGCTTTGATGGGATCGAAATATTTACCACG
TATGCGTAATATTGGTAAATTAGATGAGAAGACAAGACACGCTCTAATTCACGTTTTCG
CTGCCAAGCTTACAGCCGAGGGCCAATCAGTTGAGGAACCCAAGTCAAGATATGACGAC
GATGATGCATTCAAAAAATTGTGTGAAGACATTAACAAGTTGGACTTCGTCTTTGACGA
CAATGATAGGTCTTACTGGCTTTCAATTATTGACAACCACAACAAGGCACGTCAAGAGT
ATATGATCCACCCTGCAGAGATGGAATATATACATGTGGAACACCGAACTATTGATGGT
AAGGCTGGATTTTATTGGAGCAATGTCAATTTACCTTCCACTCTACATTCCTTCGGCAAG
GAGCATGTTGATTTCCTCGATTTGGCCACTGCTATTCACACTGCCCAGCTCATACAGAAG
TCTAGTTCAATCCCTCGATATGTTAAGGTTGCCAACTCCGGAGAAATGATCAACGAAGA
CTACCTCATATTCCAAAGCAAGCGTATTCTTAGCCACGGTAAGTTGACGGCAGGTGCCA
CTCGTACACAGAACTTGAGCACCTTTAAGGCATCTATATTTGCCAACAATGCTCCTGAAA
CAAAACAGTTCCTTAAGGATAATGAGATTAATGTCGCAAGTGATCTAGATATTGGACAC
CTCAAAGAGTATGTCTCCAATTACCCTGTTCTAGCACTACAGTTGATGTTCTTGCAAGCC
CCTCACTTTGAATTCGAATACACTGATGCCCCGGTTTTTGGGTCCTATGCCCTGGCTTGT
ATAGAATCCATGGCGGCTGGAAGGTGGGATCACCTGTTTAAAGTGGCCACGTACTGGAG
ACGCACTGGCATCTTTTCGATATTAAAGGCCGTTTCTCCAACCCTATACTCGAGGGCCAA
AACGCAGCTTGAGGACACCAAGGGAGTCACCGATGGCCTAGTGACTATGTATCTTAACA
ATATGGGCACCCGCCAGAACTTCCACTTGACTAATGATGTTTTCGATTTCAAGTACGCAG
AGAAACCTTCCCAGTCTAGGGAAGACATATATAAGGATGTTAAGGCGGTAACTTCCGAG
GATAAGGAGAAGTCATCCTTCCTCAACTTGTTCCTAAAGTGGTCTAGTGCCGATGGGCG
GAAGAACAAGCAACCATGCTACGTCTATAATGCCAGAGTGCCATTTGTTGCTGATGACA
AGGTGACGTGGAAAACTGTACCTTTGACGATATCTGGTTTTATAGTGCTGAAGACTGTCC
CTCGTTGGACCACCTCCTCTGGTACCTATTTGTTCCAGAATGCCTTCTTGGAACCCGGTG
CAGAATCTCAGACTCCTCAGCAGATGGCTGACATTACGCGTGTATTCAATGATGCTGCAT
ATGTTTTCGGAGCTGATACCATTAAGGCGCCCTTACACAACTTCTATCTTATCCTCATGA
ACCACCTTCTTCCTGGTACTGGTGACTCGTTTGATTTGAGATCATACTATGCTTGCCTTGA
AGACAAATCTTCCCCATTGCGTAGGGTCGTTTCTGAACCACTTAGTTGTGCTACTGCTTT
GCGTCTGAATTACTACAGTGCCGCTTCCCGAACTCAGGCCACTAGTGTGACTTCGAAATC
TATCCTCAAGAAGTTCATTAACAAAGGGGAATTAGACAATGAGTTATTTATGAAGTGTC
AAGAGACCTTTGCCTTAACCTGCATTGTTGGGAAAAATGATGAATTTATTTCTGCTGCTA
ATCTGAGTGACTTTGTTGAGCTAAATACAAAGCAGCGTATGTGGCAGTCCTGGTCGAGC
CTGTCAAAGACGTACAAGGAGAAAAAGGAACAGGACGAGGTTAAATTTGCTAGAACCT
TCCACCAAGCTTGGAATACTTTCACTGGAACGCTGGGCGAAGCCCTTGCTGAGAAGTTC
TATGAATGCCGCAGCCTTGAATCCCTGAAACGATTGAAAGATAAGGGTAACGCCAAATA
TCCAAATGCCTTGGATCGCGCCATTTCCGAAAAAGAAGCCCACACTGAGTGGTACAGAG
GTCTCTTATCCATACTTGGTTGGATGGCCCTTTTCGCCGGCATTTTTGCACTAGCCATTGG
CATAGGATCTTACTTCTCAGTTACTCCTGAGGGTAAATATGAAGAAGATCCAGATTATTC

TCCCGATTCTGAAGCGATGGATGGTGAGATTAATACCAAAACTCCTAATGTAGTGGTTC
CGCAGATTTTAGAGAAGGATCAAAACTTTGTGCACTTTACGTCGCCTGCTGGAGGATTCT
GGGCAATTCAAGTGTTCCAAGACGTCTTTGTGATGAACCATCACGTCTACGAGGCTTACT
CCAAGCTGGGCAACAAGCAGTTCACCTTGATCCGATCCAATTACTACCAGAGGACCCCA
GTTGCCAATCAAGTGGTTATCGATCCATATGTATTACTCGATCTACCCTCGCTCGATCAG
TGCTTCGTTTGTATGAATTTGAAGTCGCAGTATTATTCTCGGGATATTACATCAAATTTCC
TAACCGCTAAAGAGTATGAAGATTTCGTGCAGTCTGATCGTTGCCACCAGCAGCACTAC
AAGGAGAAGTGGGCTTTTGTAGCACCTGACGCGTCGCTACACAGCAGTTATCCCAGGTC
GCAGTACTGTAGCATACATACTTCCAATAGGAAAGTCCTTGCCTATGAATCTACCACCAT
CAAAGGTGATTGTGGTAAACCAACCATATTAGCCAGTGGCAATTATGCAGGTAAGATCT
GGGGCTTCCACTACGGAGCATCGGCTAACAATCTTAAGGGTAAGAGGGTAGGCTTCTCG
ACCTGCGTTCTTCAAGAAGATCTTAAGAGGGTCAAGAAACTTCTGCTCAGTTACCGTGG
CACGGCTGTTCCTTATGAAGAGGTTGAAAACCAGGCCGACCCGCTCTTAGAGGTCTGTA
AGCTACCAGATTTTGAGTGGCGACATAGTTTCAATGAGAAGAACGTATACGACTTGTCT
ATTGTTCCCCCTGCGGCGCGTGTTCACATCCCTTCAGACACGATATTTGAACCCCTCAAT
CCTGATGACCCACTTCCGTCGGATGCCCGTATTCCGGCTATATTGAGTGTTGATGACCCG
CGTTCAGATGGAGTGGATCCGGTCAAGATGGTGATTGAACAGATCAACAGTAATCCAGG
TCTCGATGCCAATAGTAGCCCTCTGTTAGGAGAATTGGATAGAGTTGCAGATGATATGG
TGAAGGATTTAACACGGTATCCCGTTTTAAATGGAGAAAAACGACCATTCACCATGGAG
GAGGCTATCTTAGGCCGCCCCGGACATCTTGGACCAATGGACTTGGATGCATCTGCTGG
CTATGAACTTGTAAAGGCCGCCCCTGGCACTCATAAATCTGATTGGGTTGTCAATGGTAG
GTTAATTGGTATTGCAAAAGCCGATTACGACAGGCGCATGACTATGATTAAGCAAGGCC
AGCCAGCATACGATCGTTACGAGGCCACTGTTGTTTTGTACCTTAAGGATGAGCTTCAAA
GACCTGAGAAGGCCCTCGTGCAGCACCGAACGCGGGGAATCTTTGCAGGTGATTTGGTA
GGTGGTGTTATATTACGCCAGATGTTTGGCCCTTTTCTTTGCTACTACTATCGCAATAGA
GTCGAGAACAACTCTGCCATAGCTACAAGCCTGTGGTCAGCAGATTATCAGGCTATTTA
CTACCATCTTTGCCATGAGTTTGGCGCTGATAGATGTGTTGACGGGGACTACAAGGGTTT
TGATACGAGTTATATTCCTTCTGTCCGTGCTAAAGCATATGAAGTGCTGTATCGTGTTGC
CAGCCTCGTATCGGAAACGCCCGCCTGCGCTTACACTACTTTGGTTAACCATGATTGTTA
TCCTACAGTGATAGCCGGTAAGTTTAGGTTTAGAGTTCACTGTCATCACTTCTCAGGCTC
CACCTTCACTAGCATCATAAATAATCTTGTGAATGAGATATACTTCCGGCTGATATTCTA
CCATCATTATCCCAATTATGCTTTCAACCAGTGTGTAAGATGTATATTCTATGGAGATGA
CCACATAGTTACTAGCAAACCTGGAGTCTTATTCGACTTCCCAATGATTCAAAAGGATAT
GGCTAAGCTTGGACAGACTTACACCTCTAGTGAGAAAGATGGTGTTAGCTTTACTTACC
ATAAGTTCGAGGAGACACAATTCTGCTCCACTAAGCCGCTTAAGTTTGGTTCCTGTTATA
TGGGTTGTCTGATACCATCGCGTGTCGAGAACTTGTTCAACTGGGCGAAGAAAACGGAT
GACGTCCAAGCGCGTTACAACCGCATCTATGCATACAAACGTATTATGGCTTCGCAGCC
TAGAGAGGAGTATCTGGCCTTTCTCGAGCGGCTCAAACGCACGTTCAGCAGTAATGACT
TACCTGCCAACACCTTTGCAGATACTCAGGTGTCAGATCGGGCTAACTGTTTTTACACCA
CCACCTTCTCATTGCTAGATCCTTATTATGATGGAGTCGTCGACGTTGAGGTTGAAGCTC
AATCAGAGGCTTTTATTGAGCCTGAAAAACCATTGATGGTCTTATCTGCTTCCGATACGC
AAACTACTCAAGCAATTGGACCGGTTGCACTTCCTGCTCTGCAACCCTATGCATGTAACG
CTGGCCCGGCTGATATTCCAAATTTAGCAGCCAATCGTGTGAAAAGAGGCAAGTGGACG
GTTTCACCATCCTACGCCGCCACAACTGTAGTAGGCAAGGTATCCATTCCTAATGAACTC
ATCACCATAGAGACTGACAAATCTGCTCAAACTATGCCGCTTAAGACCACGCAGTTGTG
TAGATTTAATATTGATTTTTCTCTAGTCGTACTTGCGAACAAAACTGTGACATGCAAGCT
TGCGCTTGTGTTTCTACCATTCCGTACCCCTGCTTCTATTGGACCTACATTCAGGTTGCAG
AGTGTCAATTGGTTACCACATATGGACATCTACCCTGATGATAACACGCTTTACACCTTT

TCTGTTCCTTTTACTTCTCCGTACAGTCTTCAGCGTACTTCCGATCTGACTTCCACGCGCT
ACTGGGGCACTCTCATTTGTGTTATGCTTACTGATGTCATCAAGCCTATCGAGCTAGGCA
CCAATCCTCTGTCGTCTTATGCTTCGATTGATATGACATATTTCTCAGGGCTCTCTCACAT
TTTCGTCACGCTGCCCAAGCCCATGTTTGAGGCGCAGGTTTTAGAGGCCGTCTCTGCCAC
CGCCCGTGAGGCCGCCGAGATATGTACATCAGTTGCCACTTATGCAGACTTGGCGGATT
CGTTGATATCTGAGTTTATGCAGGGTTGTGACGAGTTCACAGTTAACAATAAAACGGGA
GCTGTGAGCCTGCTACTCCCAAGTATGCCCAACTCCTCTGGCGTGTGTCACACAACCACT
ATGGATGTACTCCCAACTTCAAGAACTATACAGCATATCCTGCTCGGAGACCAGGAAGC
AATGTGGATTAATAAAATCTTGGAGACCCCAATGTATTTGACAACGATTAAGTGGGTTG
CCACTAAAGCGGTTGGTGAGACTCTCGCCTCTTTTGATTTGAACTCGGTTGTGTTGAGAA
CGGCTACACAAGTTCCGCTAAACATATATTTACTGAATATGTGTACCTACTATCACTGTG
ATTTCGTCTTCACTTTTGAGTTTGTCAAGACTAAGTATCATTCAGCAACCGTCCGAATTA
CTGACTCCTTTGAGCCTAAAGATATTGATCCTGCTGATGCCATGTTTTATACTACAGAGG
TTATTAATATAGACACGCAGCCCAAAATCAATCATCCTGTTTATTTCAATAACAACTTGG
AGTATCTACGCACTGTGGATGGCAACAGACTAGAGACATATGCAGCAGACCAGCGCTAT
ACCATGGGCCATACTATTCTGACCGTCGAGCAATCATTGAAAACAACCAGCTTAGTTTCT
CAGGAGATCCAGATTCTTGTCTACATCTCCTTTCACCACTTTTATGGAGTTTATCCTCGCT
CGAATTCCATTGTGCAAAAGAATGCTGAACCCGAAATCTACTTTGAGGCCCAAATGGAA
GATCCAGATCCAGCTCAGTTGCCTAATCCAGATGCGGCTGGTGAACCCCCTGCACCAAA
GAATGCGGCTTCAGACACTGGCAAGAGTGACATTCCCAAAGAGACTTTGGAGAACATGC
CAAAACCTTTGCTTAAGGCCAACGGTTTCTCACAAACTCAGACCACTGCACACCATAAC
AATTTTAAGCCGCATAACTACACAGTCGAGAACTCTGGGAAGAAGTTTGAGTATTCTCTT
TCGTCTGTGTTAGCTTTCGAGAAACGATTTTCTTGGGTTCCTCCTGAACTCATTACTAAA
GTTCGACAAGTTCAGTACCCCAGATCCTACACTATTTACACTTTTAATCCTGTCCGCATG
CTCAAATTCAAAGATATTTATGCATCTTATGCAGGTTCCTATGAGATTCGTGCTTATTTCT
CTAAGCCAACCAATATTCCTATGGCGACCGTGATACCCCTACCGCCTAATGATTTTCAAA
ATGCAAATAAGGCGGTAGAAATGGCAATCTGGCCCGGCTCTGCCATTAGCGTCACCGAA
CAAAGTAAGCTGCGAACATCTCTGGAGCCAATTAAGAATGCTCTAGAGTTGGCTACACC
TATCTCTAACGACATGTGGATGATTGACATCACTGCCCCTTTCCTTTATCAATCCAATGT
GGCTATGCTTCATGAGGGTACTACTTACTCTCTCCAGGAAGACAATCCCGTGTATATAAT
AACAGACACCACTTTTGATCTCTTTTTCCGGGTTGGTGATGATTTCTCCTACCATTTCAGA
GCAGAACCCCCAGAGTTCGTCATTGGCAAGCCTCTTGGAGTTGCCAAATTTGATGGTGTT
GTGGGGCAGTTGACTTATAATCAATAAGCATTATCCGGACCTAATGTTTTAAAAAAAAA
AAAAAAAAAAAAAAAAAAAA
